# A pilot study on the beneficial effects of additional selenium supplementation to methimazole for treating patients with Graves’ disease

**DOI:** 10.3906/sag-1808-67

**Published:** 2019-06-18

**Authors:** Bin XU, Di WU, Hong YING, Ying ZHANG

**Affiliations:** 1 Department of Nuclear Medicine, Chongqing Emergency Medical Center, Chongqing P.R. China; 2 Department of Pharmacy, Chongqing Emergency Medical Center, Chongqing P.R. China

**Keywords:** Graves’ disease, methimazole, selenium, TRAb, TPOAb, TGAb

## Abstract

**Background/aim:**

The aim of this study was to assess the effect of a combination use of methimazole (MMI) and selenium (Se) in the treatment of Graves’ disease (GD).

**Materials and methods:**

A total of 103 newly diagnosed hyperthyroidism patients were randomized to MMI and MMI + Se combination groups. After treatment for 6 months, the levels of triiodothyronine (FT3), free thyroxine (FT4), thyrotropin receptor antibody (TRAb), thyroid peroxidase antibody (TPOAb), and thyroglobulin antibody (TGAb) were observed. An in vitro culture model of thyroid cells was established and the protein expression and mRNA levels of TRAb, TPOAb, and TGAb were determined by western blot and RT-PCR.

**Results:**

A significant decrease in the levels of FT3, FT4, TRAb, TPOAb, and TGAb were observed in both groups along with a marked increase in TSH levels. Furthermore, the in vitro experiments showed that the protein expression and mRNA levels of TRAb, TPOAb, and TGAb decreased significantly. Also, compared to the MMI group, there was a greater improvement of these indices in the MMI + Se group.

**Conclusion:**

We suggest that the combined use of MMI and Se could improve the thyroid activity in patients, which may provide an effective therapy for the treatment of GD in clinical settings.

## 1. Introduction

Hyperthyroidism is an autoimmune disease that is frequently seen in the endocrinology department. It is caused by the excess release of thyroid hormone through thyroid synthesis, resulting in hypermetabolism, sympathetic nerve excitability, and finally palpitations, sweating, increases in food intake and frequency of stools, and weight loss (1). It is generally believed that immune dysfunction is involved in the occurrence and development of hyperthyroidism (2). Graves’ disease (GD) is a common autoimmune disorder of the thyroid gland, resulting in thyrotoxicosis secondary to thyroid receptor autoantibodies, which accounts for 60% to 80% of the global incidence of thyrotoxicosis (3,4). At present, the treatment of hyperthyroidism mainly includes antithyroid drugs (ATDs) (e.g., methimazole or propylthiouracil), thyroidectomy, radioactive iodine (I131), and permanently reduced thyroid function (5).

ATDs have been used as the principal treatment for GD in both Europe and China. The treatment is usually continued for 12 to 18 months, after which it is gradually tapered off (ATD treatment withdrawal) (6). Methimazole (MMI) is a common type of ATD (7–9). Its mode of action is to inhibit thyroid peroxidase, which in turn inhibits the coupling of tyrosine and iodide, and it effectively reduces the biosynthesis of thyroid hormone (10). At the same time, due to its less systemic adverse reaction to hyperthyroidism, it has been widely used as a first-line hyperthyroidism treatment in clinical settings (11,12).

GD is an autoimmune disease and is usually accompanied by immunodeficiency (13). Thus, while reducing thyroid hormones, improving the patient’s immune status should also be considered. Selenium (Se) is one of the essential trace elements in the human body and is mainly concentrated in the thyroid gland. Se can participate in the homeostasis of the thyroid hormone-dependent metabolic pathway, which plays an important role in maintaining the integrity of the cell membrane, iodine metabolism, and normal thyroid function. At the same time, it can also eliminate free radicals and enhance immune function systemically.

Although MMI and Se both have a significant effect on the immune system as well as on the reduction of autoantibodies, their combined clinical efficacy has seldom been reported. We hypothesize that the addition of Se supplementation to the usual treatment with MMI will lead to a better clinical outcome and improve the quality of life of patients with GD. Through clinical and in vitro studies, we expect to provide a theoretical basis for the clinical treatment of GD with a combination of MMI and Se.

## 2. Materials and methods

### 2.1. Subjects

A total of 103 newly diagnosed patients with hyperthyroidism were consecutively enrolled in the study at our hospital from January 2016 through June 2017. The inclusion criteria were as follows: 1) to have met the diagnostic criteria for GD according to the Chinese Diagnosis and Treatment of Thyroid Diseases guidelines; 2) no prior ATD use or contraindications for treatment with ATDs before admission; 3) patients with no comorbid diseases such as heart failure, kidney, or liver disease; 4) a low level of thyroid-stimulating hormone (TSH) and a high level of free thyroxine (FT4); 5) good compliance with medical advice. The exclusion criteria were: 1) being under 18 years of age; (2) severe heart or liver dysfunction; (3) presence of other endocrine system and blood system lesions; (4) pregnancy or lactation; (5) recent use of immunosuppressive agents and ATDs. This study was conducted with approval from the Ethics Committee of Chongqing Emergency Medical Center. Written informed consent was obtained from all participants.

### 2.2. Treatment

Patients were randomly divided into two groups, one group receiving MMI and the other the MMI + Se combination. Patients in the MMI group were given a placebo tablet (twice a day) and a 30 mg MMI tablet (Merck KGaA, Darmstadt, Germany) daily for 6 months. Patients in the MMI + Se group received a 30 mg MMI tablet daily and 300 µg Se (twice a day) (Ling Tai Pharmaceutical Limited by Share Ltd., Hei Longjiang, China) for 6 months. The thyroid function of the patients was periodically reviewed every month and the doses of MMI were gradually reduced based on the recovery of thyroid function until the minimal maintenance treatment dose was reached. At the same time, the corresponding symptomatic treatment was implemented according to the patient’s own condition.

### 2.3. Observation indices

A total of 5 mL of venous blood was collected from each patient before treatment, and this was repeated during follow-up. The concentrations of free triiodothyronine (FT3), free thyroxine (FT4), TSH, thyrotropin receptor antibody (TRAb), thyroid peroxidase antibody (TPOAb), and thyroglobulin antibody (TGAb) were measured by chemiluminescent methods according to the manufacturer’s instructions (Jiuding Co., Ltd., Tianjing, China).

### 2.4. Thyroid tissue collection and cell cultures

The thyroid tissues of patients with hyperthyroidism (n = 10, TP) and normal tissues around benign thyroid adenoma (n = 10, NO) were obtained. None of the patients had been treated previously with ATDs. The thyroid tissues were minced and placed directly in plastic culture dishes, and a 10-fold volume of sucrose solution (0.32 mol/L) was added and then homogenized at 4 °C. After centrifugation, the supernatant was taken and sucrose solution (2 mL, 0.80 mol/L) was added again. Centrifugation was performed again and the precipitation was collected. An equivalent volume of trypsin solution (0.25%, pH 8.0) was added and the mixture was incubated at 37 °C with 5% CO2 for digestion. After the single-cell suspension was made, cells were cultured in high-glucose Dulbecco’s modified Eagle’s medium (DMEM) (HyClone, Logan, UT, USA) supplemented with 10% (v/v) fetal bovine serum (FBS), 100 U/mL penicillin, and 100 mg/mL streptomycin and were maintained in a humidified incubator with 5% CO2 at 37 °C.

### 2.5. Cell viability analysis

The 3-(4,5-dimethyl-2-thiazolyl)-2,5-diphenyl-2-H-tetrazolium bromide (MTT) method was performed to evaluate the effect of MMI and Se on cell viability. Briefly, thyroid tissue cells were seeded into 96-well culture plates (5 × 105 cells/well) and treated with different concentrations of MMI or Se (0–40 µM) for 24 h. Also, effect of time on cell viability was observed (6–48 h). After treatment, the cells were incubated with 5 mg/mL MTT solution for 4 h at 37 °C. The medium was then removed and dissolved in 150 µL of dimethyl sulfoxide (DMSO) for another 10 min, and analyzed spectrophotometrically. The absorbance of the dye was measured at 570 nm using a microplate reader (BioTek, Doraville, GA, USA).

### 2.6. Detection of cell apoptosis by flow cytometry

To evaluate the effect of MMI and Se on the apoptosis of cultured thyroid tissue cells, an Annexin V-fluorescein isothiocyanate (FITC) kit was used to detect apoptotic cells. After treatment with MMI or Se, the cells were harvested and washed with PBS and resuspended in binding buffer. Then the cells were incubated with FITC and propidium iodine (PI) for 15 min in the dark. Finally, cell apoptosis was detected with a flow cytometer (Becton and Dickinson Influx, USA).

### 2.7. Detection of TRAb, TPOAb, and TGAb proteins

After treatment with MMI and/or Se for 24 h, the thyroid cells were harvested and lysed in a RIPA and PMSF mixture (RIPA:PMSF = 99:1). The total protein concentrations were determined by using a bicinchoninic acid kit (Beyotime, Jiangsu, China). Then equal amounts of proteins (50 µg/lane) were separated by SDS-PAGE and transferred to PVDF membranes. The membranes were incubated with different primary antibodies, rabbit anti-TRAb (1:400, Boster, Wuhan, China), rabbit anti-TPOAb (1:400, Boster), or rabbit anti-TGAb (1:400, Boster), overnight at 4 °C. The immunoblots were then incubated with antirabbit IgG-horseradish peroxidase (1:4000, Boster) as a secondary antibody followed by ECL detection (Millipore, Billerica, MA, USA).

### 2.8. Measurement of TRAb, TPOAb, and TGAb mRNA levels

After treating with MMI and/or Se, the total RNA (1 µg) of cells was isolated and reverse-transcribed into cDNA according to the manufacturer’s instructions (TaKaRa, Dalian, China). The PCR reaction was carried out using the SYBR Premix Ex TaqTMII Kit (TaKaRa) according to the manufacturer’s instructions. The primers of the reaction system were designed and synthesized by Sangon Biotech (Shanghai, China) as follows: TRAb forward primer: 5′-GCAGCCAGGCACCAGAACATC-3′ and reverse primer: 5′-TGCCAACAGCAGCCAAGAAGG-3′ (185 bp); TGAb forward primer: 5′-TGCTGGCCTGGACCTTCCTTC-3′ and reverse primer: 5′-CGGCGGCAGCTTGGAACATAG-3′ (177 bp); TPOAb forward primer: 5′-CGCTCGCTGTGCTGTCTGTC-3′ and reverse primer: 5′-GTGGCGTACATGGCGGTGTC-3′ (163 bp). All PCRs were performed in triplicate. GAPDH expression was used for standardization, and the relative expression analysis was assessed by the 2–ΔΔCt method.

### 2.9. Statistical analysis

All the data were presented as mean ± SD and analyzed using SPSS 22.0 (IBM Corp, Armonk, NY, USA). Student’s t-test and the chi-square test were employed for multiple group comparisons. One-way ANOVA was performed for repeated measures and the Student–Newman–Keuls test was used for unequal sample numbers. P < 0.05 was considered to be statistically significant.

## 3. Results

### 3.1. Comparison of thyroid function and MMI therapy

A total of 103 subjects were originally enrolled in this trial, 95 (92.23%) of whom completed the trial. No significant differences were observed in age, sex, or levels of FT3, FT4, TSH, TRAb, TPOAb, and TGAb between the two groups before treatment (P > 0.05, Table). After 6 months of treatment, the levels of FT3, FT4, TRAb, TPOAb, and TGAb had significantly decreased in both groups, and TSH levels had significantly increased (P < 0.05). Intriguingly, except for TSH levels, the MMI + Se group showed substantially more improvement in all of the measurements than the MMI group (P < 0.05, Table).

**Table:** The baseline parameters and clinical efficacy of the two groups (n = 95).

Groups	MMI		MMI + Se		F/c2	P
	Before	After	Before	After		
Sex						
Male	19		14		0.393	0.531
Female	31		30			
Age	40.20 ± 12.63		38.89 ± 11.59		0.252	0.602
FT3	28.42 ± 14.76	13.39 ± 7.41*	27.93 ± 10.82	5.29 ± 1.02**	12.727	0.003
FT4	66.14 ± 26.52	16.59 ± 3.34**	70.97 ± 29.12	9.29 ± 4.27**	7.897	0.037
TSH	0.006 ± 0.001	2.11 ± 0.32**	0.004 ± 0.002	2.30 ± 0.54**	3.099	0.773
A-TG	772.69 ± 166.16	440.99 ± 104.27*	778.42 ± 199.90	366.37 ± 78.88*	57.752	<0.001
A-TPO	353.21 ± 36.71	128.28 ± 36.95	321.36 ± 30.02	95.22 ± 23.68*	0.848	<0.001
TRAb	21.01 ± 11.69	5.27 ± 3.46**	20.09 ± 12.51	3.33 ± 1.43*	3.953	0.004

Data are represented as mean ± SD. *P < 0.05 and **P < 0.001 versus patients before treatment.

### 3.2. Effects of MMI and Se on the cell viability of thyroid cells

**Figure 1:**
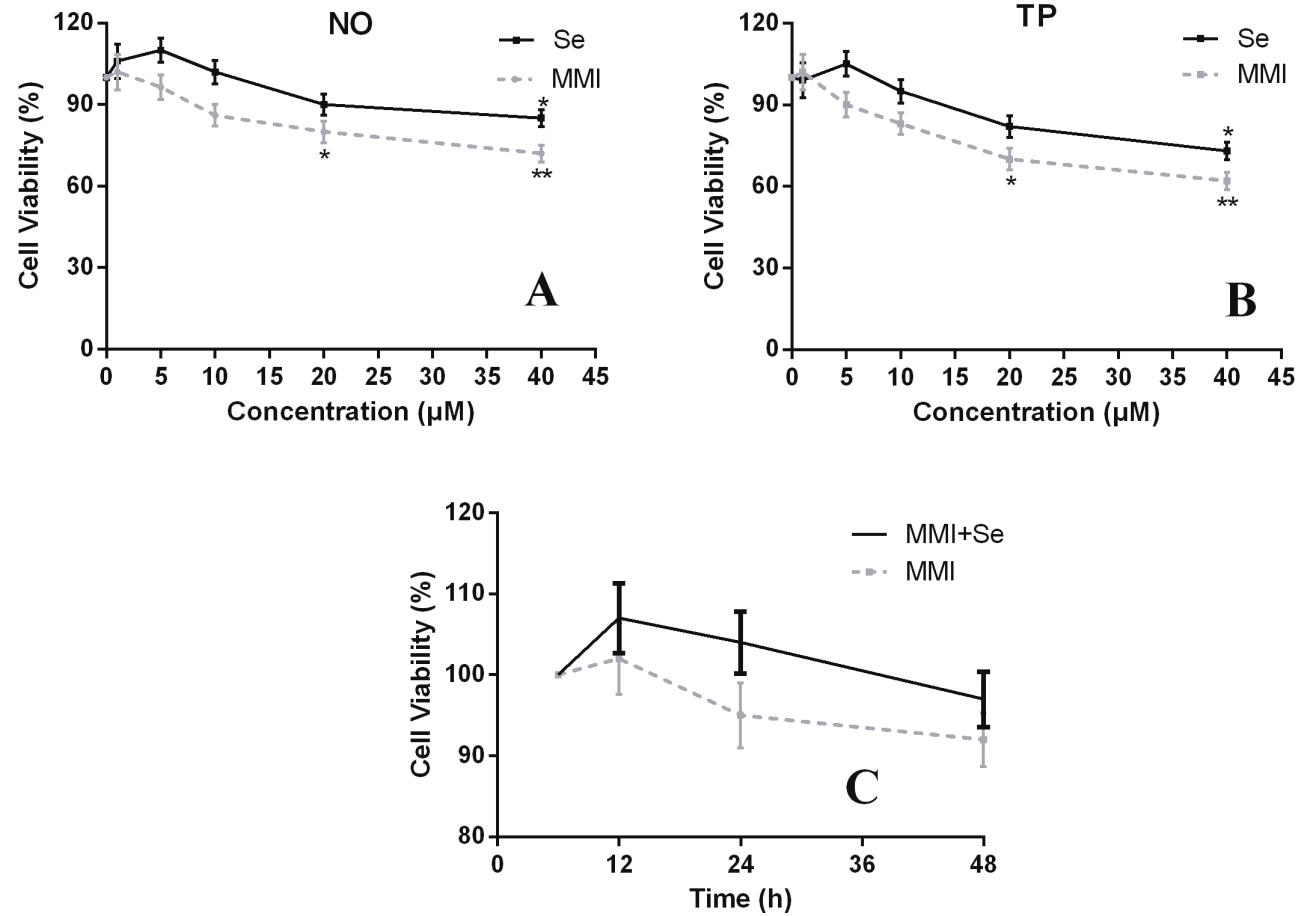
The effects of MMI and Se on the cell viability of thyroid cells. Thyroid cells were treated with different concentrations of MMI and/or Se and cell viability was observed by MTT. (A) NO group; (B) TP group; (C) cells treated with MMI and/or Se for 6, 12, 24, and 48 h. NO: Normal thyroid tissue; TP: hyperthyroidism tissue. The data are represented as mean ± SD. *P < 0.05 and **P < 0.001 versus untreated group.

MTT was performed to evaluate the effect of MMI and/or Se on cell viability. After treatment with MMI and/or Se for 24 h, the cell viability decreased with increasing drug concentrations, especially when MMI reached 20 µM and Se reached 40 µM (P < 0.05, Figures 1A and 1B). Moreover, treatment for 6, 12, 24, and 48 h was also performed at each of these concentrations, and there was no significant change in cell viability (Figure 1C).

### 3.3. Effects of MMI and Se on apoptosis

**Figure 2:**
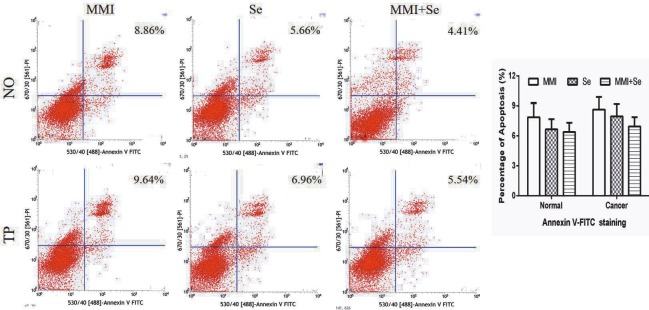
The effects of MMI and Se on the level of apoptosis. Cells were treated with MMI (20 μM) and/or Se (40 μM) for 24 h. Images represent cell apoptosis and the percentage of total apoptotic cells by Annexin V-FITC/PI staining assay. The data are represented as mean ± SD.

To measure the rate of cell apoptosis and evaluate the safety of MMI and Se, Annexin V/PI staining for flow cytometric analysis was used. As depicted in Figure 2, our data showed that no significant rate of apoptosis was found after treatment with MMI, Se, or MMI + Se.

### 3.4. Effects of MMI and Se on the expression of TRAb, TPOAb, and TGAb

**Figure 3:**
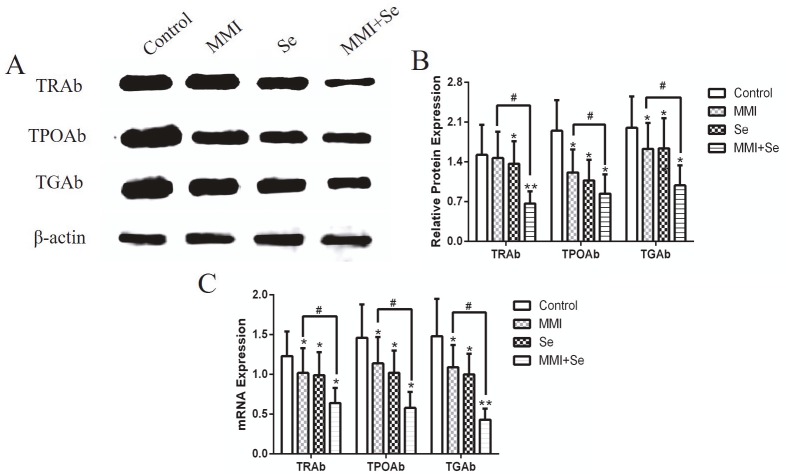
Western blot and RT-PCR analysis of the effects of MMI and Se on the expression of TRAb, TPOAb, and TGAb. Cells were treated with MMI (20 μM) and/or Se (40 μM) for 24 h. (A) Protein expressions of TRAb, TPOAb, and TGAb. (B) Representative images of protein expressions of TRAb, TPOAb, and TGAb. (C) Representative images of mRNA expression of TRAb, TPOAb, and TGAb. The data are represented as mean ± SD of three experiments. *P < 0.05 and **P < 0.001 versus untreated control group; #P < 0.05 versus untreated control group.

Western blot and RT-PCR were used to detect the protein expression or mRNA levels of TRAb, TPOAb, and TGAb after the treatment of cells with MMI and/or Se for 24 h. As shown in Figure 3, the protein expressions of TRAb, TPOAb, and TGAb were significantly decreased when compared with untreated cells. Moreover, the protein expressions of these proteins were significantly more decreased than those of the MMI treatment group (P < 0.05). The RT-PCR results were basically consistent with the western blot results.

## 4. Discussion

Hyperthyroidism is a disease of the thyroid gland caused by excessive secretion of thyroid hormones and is classified as a multiple endocrine system disease (1,14). Patients with hyperthyroidism are prone to relapse and are greatly affected by genetic factors (15), making the disease difficult to cure. Graves’ disease is a common autoimmune disease of the thyroid gland and a leading cause of hyperthyroidism worldwide (16). The detection of TSH receptor autoantibodies can be used for a diagnosis of GD and to explain the abnormal increase of thyroid hormone concentration in blood (16). Through treatment with MMI and/or Se, the levels of FT3 and FT4 were significantly reduced (P < 0.05). Also, the levels of TSH increased remarkably. Together with these results, a satisfactory clinical effect was obtained in this study.

MMI is one of the most common kinds of ATDs, which can inhibit the synthesis of peroxidase and limit the synthesis of TSH and FT3, thus improving the level of thyroid hormone (10). Numerous studies have shown that the concentration of drugs in the thyroid gland is closely related to the daily dose of the drug (14,17,18). Selenium is a necessary trace element of key importance for homeostasis of the human system, especially for the normal functioning of the immune system (19–21). There are many hypotheses about the protective role of selenium in thyroid diseases. At present, it is presumed that Se supplementation can reduce the concentration of antithyroid antigen antibodies, protect the thyroid from oxidative stress, and optimize the synthesis and transport of TH by inducing selenoprotein synthesis (22). The immune system depends on adequate intake of dietary selenium, and this nutrient plays its biological role mainly by its incorporation into selenoproteins (23). It has been assumed that a low level of Se can cause immune dysfunction, induce hypothyroidism, and trigger insufficiency of the free radical scavenging system, eventually leading to the death of thyroid immune cells and the initiation of chronic inflammation (24).

MMI and Se have shown good results in treating hyperthyroidism, and the mechanisms of treatment are basically the same. Both drugs can inhibit the activity of thyroid peroxidase and prevent iodine ions from oxidizing to active iodine. By hindering the tyrosine condensation reaction of iodization and inhibiting the synthesis of FT3 and FT4, they can both have antithyroid activity. After 6 months of treatment, our results showed that the levels of FT3 and FT4 in the MMI + Se group were significantly lower than those of the MMI group, and the level of TSH was higher in the MMI + Se than in the MMI group (P < 0.05), indicating that the effect of combination therapy on hyperthyroidism is more prominent than that of MMI alone. 

The toxic effects of MMI and Se on cells were detected by MTT and flow cytometry. The results showed that when the concentrations of MMI and Se reached 20 µM and 40 µM, respectively, the cells began to exhibit apoptosis, indicating that the two drugs are safe to use. At the same time, some studies have demonstrated the benefits and safety of Se supplementation in the treatment of hyperthyroidism (25–29). In a previous study, the levels of FT3 and FT4 in patients with GD decreased rapidly after treatment with MMI, and with the addition of Se, TSH rose significantly. Our results are consistent with those of Vrca et al. (30) to some extent. 

TRAb is an antibody specific to humans and is the main direct cause of hyperthyroidism. TRAb can be used to predict the recurrence of GD. A decrease in TRAb antibody titer can be seen as a sign of improvement during the treatment of GD. At the same time, the lack of TRAb can be used as an indicator to reflect the remission of GD patients during the late treatment period (12,31). TPOAb and TGAb are autoantibodies specific to thyroid disease, and increasing antibody titers were observed in the early stages of GD. The antibody titer gradually decreased with remission of the disease (32,33). Previous studies showed that Se therapy could reduce the level of TPOAb and indicated that it has beneficial effects on immune activity (5,34,35). Through in vivo and in vitro experiments, our results showed that the levels of TRAb, TPOAb, and TGAb were markedly lower in both serum and cells. Moreover, the combination group had lower levels of antibodies than the MMI group, showing that the combined use of MMI and Se could significantly improve immune function in the patients. Further studies will be focused on the clinical effect of Se on GD.

In conclusion, clinical and in vitro experiments were conducted to evaluate the therapeutic effect of the combination of MMI and Se on hyperthyroidism, and our results found that the group that received the combination treatment had lower levels of FT3 and FT4 and lower TRAb, TPOAb, and TGAb expressions than the MMI group. Furthermore, higher TSH levels were observed after treatment with MMI + Se. Thus, our results point to a simple and safe strategy for treating hyperthyroidism.
